# Restored Fyn Levels in Huntington’s Disease Contributes to Enhanced Synaptic GluN2B-Composed NMDA Receptors and CREB Activity

**DOI:** 10.3390/cells11193063

**Published:** 2022-09-29

**Authors:** Lígia Fão, Patrícia Coelho, Ricardo J. Rodrigues, A. Cristina Rego

**Affiliations:** 1 Center for Neuroscience and Cell Biology (CNC), University of Coimbra (Pólo I), Rua Larga, 3004-504 Coimbra, Portugal; 2 Faculty of Medicine, University of Coimbra (Pólo III), 3000-548 Coimbra, Portugal; 3 Institute for Interdisciplinary Research (IIIUC), University of Coimbra (Pólo II), 3030-789 Coimbra, Portugal

**Keywords:** fyn kinase, mutant huntingtin, NMDA receptors, Huntington’s disease, CREB, post-synaptic density

## Abstract

*N*-methyl-D-aspartate receptors (NMDARs) are important postsynaptic receptors that contribute to normal synaptic function and cell survival; however, when overactivated, as in Huntington’s disease (HD), NMDARs cause excitotoxicity. HD-affected striatal neurons show altered NMDAR currents and augmented ratio of surface to internal GluN2B-containing NMDARs, with augmented accumulation at extrasynaptic sites. Fyn protein is a member of the Src kinase family (SKF) with an important role in NMDARs phosphorylation and synaptic localization and function; recently, we demonstrated that Fyn is reduced in several HD models. Thus, in this study, we aimed to explore the impact of HD-mediated altered Fyn levels at post-synaptic density (PSD), and their role in distorted NMDARs function and localization, and intracellular neuroprotective pathways in YAC128 mouse primary striatal neurons. We show that reduced synaptic Fyn levels and activity in HD mouse striatal neurons is related to decreased phosphorylation of synaptic GluN2B-composed NMDARs; this occurs concomitantly with augmented extrasynaptic NMDARs activity and currents and reduced cAMP response element-binding protein (CREB) activation, along with induction of cell death pathways. Importantly, expression of a constitutive active form of SKF reestablishes NMDARs localization, phosphorylation, and function at PSD in YAC128 mouse neurons. Enhanced SKF levels and activity also promotes CREB activation and reduces caspase-3 activation in YAC128 mouse striatal neurons. This work supports, for the first time, a relevant role for Fyn protein in PSD modulation, controlling NMDARs synaptic function in HD, and favoring neuroprotective pathways and cell survival. In this respect, Fyn Tyr kinase constitutes an important potential HD therapeutic target directly acting at PSD.

## 1. Introduction

Huntington’s Disease (HD) is an autosomal dominant progressive neurodegenerative disorder that affects mainly the striatum (caudate and putamen) and later the cortex [1]. HD is characterized by psychiatric and behavioral disturbances, such as motor impairment, and psychiatric symptoms such as obsessive-compulsive disorder, depression and/or anxiety, cognitive decline and weight loss [2,3]. HD is caused by an abnormal expansion of cytosine-adenine-guanine (CAG) repeat at the *HTT* gene, encoding for mutant huntingtin (mHTT) retaining a polyglutamine (polyQ) extension at the N-terminus [4]. mHTT has been associated with protein conformational changes, aggregation, and abnormal protein–protein interactions [2], which cause cytotoxicity, evidenced through changes in gene transcription, synaptic dysfunction, *N*-methyl-D-aspartate receptors (NMDARs) overactivation, decreased mitochondrial calcium (Ca^2+^) handling and organelle dysfunction, and increased oxidative events, leading to neuronal death [4]. NMDARs involvement in HD pathogenesis is not completely clear. HD-affected medium spiny neurons (MSNs) show altered NMDAR currents as well as augmented ratio of surface to internal GluN2B-containing NMDARs, with augmented accumulation at extrasynaptic sites [5]. Nonetheless, the mechanism underlying this altered NMDARs localization is largely unknown.

NMDARs are important postsynaptic receptors that contribute to normal synaptic function and cell survival. Activation of synaptic NMDARs is associated with augmented cAMP response element-binding protein (CREB)-dependent gene expression. CREB is a signal-regulated transcription factor, important for neuronal survival with important roles in several processes, namely synaptic plasticity, neurogenesis, learning, and memory. Conversely, extrasynaptic NMDARs activation promotes cell death pathways linked to dendritic blebbing, loss of mitochondrial membrane potential, and CREB shut-off pathway, with blockade expression of brain-derived neurotrophic factor (BDNF), a neurotrophin relevant for survival of striatal neurons (e.g., 15). Altered NMDAR function has been related to HD, as documented by previous studies. Milnerwood and colleagues showed that YAC128 HD mouse striatum presented increased extrasynaptic NMDAR expression and currents and reduced nuclear CREB activation, which was reversed after NMDAR inhibition with memantine [5].

Interestingly, intracellular mechanisms involved in HD pathogenesis, as altered striatal glutamatergic synapses and mitochondrial dysfunction linked to redox changes, are also relevant neuronal pathways modulated by c-Src and Fyn, two ubiquitous proteins belonging to the Src kinase family (SKF), predominantly located in synaptic membranes [6]. c-Src and Fyn are broadly expressed in the Central Nervous System (CNS), being enriched in striatal neurons [7], and have been implicated in brain neuronal development, transmission, synaptic activity, and plasticity in mammalian CNS, being both kinases activated by hydrogen peroxide (H_2_O_2_) [8]. Importantly, Fyn can be found in the postsynaptic density (PSD) concomitantly with components of the NMDAR complex [9]. Fyn interacts and phosphorylates NMDARs and postsynaptic scaffold proteins, as PSD95, to regulate synaptic transmission and plasticity [9]. Moreover, Fyn phosphorylates NMDAR GluN2B subunit at Tyr1472, increasing receptor retention at the synapse, thus controlling synaptic plasticity [10,11]. Several studies evidenced depressed synaptic transmission in HD. Importantly, there is a clear evidence for altered dendrite morphology in MSNs of HD patients, showing recurved endings and appendages, altered spine density and abnormalities in dendritic spine size and shape [12]. Moreover, Murmu and coworkers showed that mHTT in R6/2 mice causes a progressive loss of persistent-type spines, important for neuronal circuitry and long-term memory in the brain [13,14]. Recently, we showed that c-Src/Fyn activation and total protein levels are reduced in several human and mouse HD models mainly due to autophagy degradation [15]. Moreover, restoration of active SKF levels improves mitochondrial morphology and function, namely through improved mitochondrial transmembrane potential, mitochondrial basal respiration, and ATP production, diminishing ROS levels [15]. Additionally, YAC128 mouse striatum revealed increased synaptic activity of striatal-enriched protein tyrosine phosphatase (STEP), correlating with decreased GluN2B phosphorylation at Tyr1472, reducing synaptic NMDARs by facilitating their movement to extrasynaptic sites [16].

Based on these findings, in this study we aimed to explore the impact of HD-mediated altered Fyn levels on synaptic versus non-synaptic NMDARs function and localization, as well as intracellular neuroprotective pathways. Our data reveal that SKF activation is important for normal synaptic function and neuronal survival in neurons expressing mHTT by contributing for synaptic NMDARs presence and function. Thus, synaptic modulation of Src/Fyn activation/levels may constitute a therapeutic potential target in HD.

## 2. Materials and Methods

### 2.1. YAC128 Mice

YAC128 mice, previously described by Slow and co-authors (2003) [17], express full- length mutant HTT with 128 CAG repeats from a yeast artificial chromosome (YAC) transgene (RRID: MGI_MGI:3613515). YAC128 (line HD53) and wild-type mice were housed in the animal facility of the Center for Neuroscience and Cell Biology and Faculty of Medicine at the University of Coimbra (Coimbra, Portugal), under controlled temperature (22–23 °C) and a 12 h light/12 h dark cycle with lights on at 07:00 h. Food and water were available ad libitum throughout the experiment. Animal experiments in this study were performed in accordance with the European Community directive (2010/63/EU) and protocols approved by the Faculty of Medicine, University of Coimbra (ORBEA_189_2018/11042018). All efforts were made to minimize animal suffering and to reduce the number of animals used. Animals were used at 3, 6, and 12 months of age.

### 2.2. Primary Striatal Neurons from YAC128 Mice

Primary striatal neurons were prepared as described previously [18], with some minor modifications. At 16 days of gestation, pregnant female mice were sacrificed by cervical dislocation following anesthesia using (RS)-2-chloro-2-(difluoromethoxy)-1,1,1-trifluoro-ethane. Striata were dissected out from fetal mice and cells were separated by mechanical digestion using a pipette in Ca^2+^- and Mg^2+^-free Hank’s balanced salt solution containing 137 mM NaCl, 5.36 mM KCl, 0.44 mM KH_2_PO_4_, 0.34 mM Na_2_HPO_4_.2H_2_O, 5 mM glucose, 1 mM sodium pyruvate, and 10 mM HEPES, at pH 7.2. Cells were plated at a density of 8.4 × 10^4^ cells/cm^2^ in poly-D-lysine-coated 6-well or 96-well plates, and at a density of 4.2 × 10^4^ cells/cm^2^ in poly-D-lysine coated glass coverslips for immunocytochemistry. Cells were cultured for 12 days in Neurobasal medium supplemented with 2% B27, 0.5 mM glutamine and 0.12 mg/mL gentamicin, at 95% air and 5% CO_2_. To reduce glia growth, 10 μM of the mitotic inhibitor 5-fluorodeoxyuridine (5-FDU, Sigma, #F0503, St. Louis, MI, USA) was added to the culture after 72 h in culture. One half of the medium was changed with fresh medium without 5-FDU at day 7.

### 2.3. Constructs and Neuron Transfection

Cells were transfected with pLNCX chick SKF^Y527F^ (Addgene, plasmid #13660), empty pLNCX vector, and GFP (Origene, Rockville, MD, USA, GFP; #PS100010). Empty vector pLNCX was obtained from the SKF^Y527F^ plasmid using the restriction enzyme digestion ClaI (BioLabs, #Ro197L) according to manufacturer’s protocol. The result of digestion was visualized in 1% agarose gel; the band corresponding to the empty vector was cropped and DNA was extracted using the NucleoSpin^®^ Gel and PCR Clean-up (Macherey-Nagel, #740609) according to the manufacturer’s protocol. Then, the blunt end and cohesive end termini of the resulting empty vector were joined using T4 DNA Ligase enzyme (BioLabs, #M0202S). Transfection was performed at 8 DIV using the Ca^2+^ phosphate precipitation method. Briefly, plasmid was diluted in TE (1 mM Tris-HCl pH 7.3, 1 mM EDTA), followed by the addition of CaCl_2_ (2.5 M CaCl_2_ in 10 mM HEPES, pH 7.2). The DNA solution was carefully added to 2× HEBS (12 mM dextrose, 50 mM HEPES, 10 mM KCl, 280 mM NaCl, and 1.5 mM Na_2_HPO_4_.2H_2_O, pH 7.2) while bubbling air through the solution with a micropipette. The mixture was then incubated for 25 min at room temperature. The precipitates were added dropwise to the coverslips in Neurobasal medium and incubated for 80 min, at 37 °C. The DNA–Ca^2+^-phosphate precipitates were dissolved in freshly made dissolution medium (Neurobasal medium with 20 mM HEPES, pH 6.8) and incubated for 7 min at room temperature. The transfected neurons were then washed with Neurobasal medium and transferred back to their original dishes containing conditioned culture medium.

### 2.4. Sample Preparation and Western Blotting

Total extracts were obtained from primary striatal neurons or striatal or cortical brain areas from YAC128 mice at 3, 6, or 12 months of age. The cells were scraped and brain areas suspended in Ripa buffer (containing 150 mM NaCl, 50 mM Tris HCl, 5 mM EGTA, 1% Triton X-100, 0.1% SDS, 0.5% deoxycholate, pH 7.5) supplemented with 100 nM okadaic acid, 1 mM PMSF, 25 mM NaF, 1 mM Na_3_VO_4_, 1 mM DTT, and 1 μg/mL protease inhibitor cocktail (chymostatin, pepstatin A, leupeptin and antipain). Total homogenates were lysed in an ultrasonic bath (UCS 300-THD; at heater power 200 W and frequency 45 kHz) during 10 s and centrifuged at 4 °C for 10 min at 20,800× *g* to remove cell debris. The supernatant was collected, and protein content was determined using the Bio-Rad protein assay reagent based on the Bradford dye-binding procedure (Bio-Rad, Hercules, CA, USA). Then, protein extracts were denatured with 6× concentrated loading buffer (containing 300 mM Tris-HCl pH 6.8, 12% SDS, 30% glycerol, 600 mM DTT, 0.06% bromophenol blue) at 95 °C, for 5 min. Equivalent amounts of protein samples (15 μg–30 μg) were separated by 8–12% SDS-PAGE and electroblotted onto polyvinylidene difluoride (PVDF) membrane (Millipore, Burlington, MA, USA). Membranes were further blocked with 5% (*w*/*v*) BSA (Santa Cruz Biotechnology, Santa Cruz, CA, USA) in Tris Buffered Saline ((TBS) containing 250 mM, 150 mM NaCl, pH7.6) plus 0.1% Tween 20 before incubation with the specific antibody against: GluN2A (1:1000, Millipore, Burlington, MA, USA, #07-632), p(Tyr1472)GluN2B (1:1000, Cell Signaling, Danvers, MA, USA, #4208S), GluN2B C-terminal (1:1000, Millipore, Burlington, MA, USA, MAB #5778), βActin (1:5000, Sigma, St. Louis, MI, USA, #A5316) overnight, at 4 °C. βActin was used as a control of protein loading of total extracts. An anti-rabbit (1:20,000; Thermo Fisher Scientific, Waltham, MA, USA, #31340) or anti-mouse (1:20,000; Thermo Fisher Scientific, Waltham, MA, USA #31320) IgG secondary antibodies conjugated to the alkaline phosphatase, prepared in 1% (*w*/*v*) BSA in TBS-T, were used for 1 h at room temperature. Immunoreactive bands were visualized by alkaline phosphatase activity after incubation for 5 min with ECF reagent (GE Healthcare Bio-Sciences, Piscataway, NJ, USA) on Bio-Rad ChemiDoc Touch Imaging System (Bio-Rad, Hercules, CA, USA) and quantified using Image Lab analysis software (Bio-Rad, Hercules, CA, USA).

### 2.5. Immunocytochemistry

To assess Fyn, P(Tyr416)SKF, GluN2B, or P(Tyr1472)GluN2B levels, primary neurons from WT and YAC128 mice were cultured on glass coverslips. When applicable, 24 h after transfection, cells were fixed with 4% paraformaldehyde (pre-warmed at 37 °C) for 20 min and permeabilized in 0.1% Triton X-100 in PBS for 2 min. Then, cells were blocked for 1 h at room temperature with 3% (*w*/*v*) BSA in PBS and further incubated with the primary antibody prepared in blocking solution, overnight, at 4 °C (antibodies referred above, and against CREB (Abcam, Cambridge, UK, #ab32515), P(Ser133)CREB (Cell Signaling, Danvers, MA, USA, #9196), PSD-95 (Thermo Scientific, Waltham, MA, USA #7E3-1B8), and caspase3-cleaved (Cell Signaling, Danvers, MA, USA #9664), at 1:200). Cells were then washed with PBS and incubated with the adequate secondary antibody (Alexa Fluor-594 goat anti-rabbit (Invitrogen, Carlsbad, CA, USA #R37117), Alexa Fluor-488 donkey anti-rabbit (Invitrogen, Carlsbad, CA, USA #R37118) and Alexa Fluor-488 donkey anti-mouse (Invitrogen, Carlsbad, CA, USA #R37114)) at 1:300 in blocking solution for 1 h at room temperature. Nuclei were stained with 1 μg/mL Hoechst 33342 in PBS (Invitrogen, Carlsbad, CA, USA) for 10 min and coverslips were mounted using Mowiol 40-88 (Sigma Chemical, St. Louis, MI, USA). Confocal images were obtained using a Plan-Apochromat/1.4NA 63x lens on an Axio Observer.Z1 confocal microscope (Zeiss Microscopy, Oberkochen, Germany) with Zeiss LSM 710 software.

### 2.6. Electrophysiological Recordings

NMDA-induced currents were recorded in transfected primary striatal neurons (DIV 11) at −60 mV by whole-cell patch clamping using an AxonPatch 200B amplifier (Molecular Devices, San Jose, CA, USA). The borosilicate glass micropipettes used had a resistance of 4–6 MΩ and were filled with the following internal solution (in mM): CsMeSO_4_ 130, CsCl 10, CaCl_2_ 0.5, EGTA 5, HEPES 10, and NaCl 10 (pH 7.3 adjusted with CsOH). Cells were perfused with extracellular solution containing 140 mM NaCl, 2.5 mM KCl, 1.8 mM CaCl_2_, 10 mM HEPES, and 15 mM glucose supplemented with 10 μM glycine (pH 7.4 adjusted with NaOH). NMDA (100 μM) was diluted in the extracellular solution and rapidly perfused with a six-channel perfusion valve control system VC-77SP/perfusion fast-step SF-77B (Warner Instruments, Hamden, CT, USA). All experiments were performed at RT (22–25 °C). The currents were filtered at 1 kHz (4-pole low-pass Bessel filter) and digitized at a sampling rate of 10 kHz to a personal computer and analyzed with pClamp 10.7 software (Molecular Devices, San Jose, CA, USA).

### 2.7. Measurement of Intracellular Calcium Levels

Twenty-four hours after transfection with empty or empty+ SKF^Y527F^, primary striatal neurons were incubated in experimental media (in mM: 132 NaCl, 4 KCl, 1 CaCl_2_, 1.2 NaH_2_PO_4_.H_2_O, 1.4 MgCl_2_, 6 Glucose, 10 HEPES, pH 7.4) plus 2 μM Fluo4-AM (Thermo Fisher Sci., #F14201) for 45 min, at 37 °C. Cells were then washed, and the experiment was recorded in experimental media without Mg^2+^ and supplemented with glycine (20 μM) and serine (30 μM). Fluo4 fluorescence was monitored before and after exposure to 100 μM NMDA in primary striatal neurons from WT and YAC128 mice, using an Axio Observer Z1 system, a fully motorized inverted widefield microscope (Zeiss, Jena, Germany) equipped with a large stage incubator for temperature and humidity control and EC plan-neofluar/1.3NA 63x lens. Fluo4 fluorescence was imaged along time at 494 nm excitation and 506 nm emission, respectively. Fluorescence intensities were calculated using Fiji software (Zurich, Switzerland).

### 2.8. Statistical Analyses

Data were analyzed by using Excel (Microsoft, Seattle, WA, USA) and GraphPad Prism 8 (GraphPad Software, San Diego, CA, USA) software, and are expressed as the mean ± S.E.M. of the number of independent experiments or cells indicated in figure legends. Comparisons among multiple groups were performed by one-way ANOVA followed by the Bonferroni or Dunnett’s nonparametric Multiple Comparison post-hoc tests or by two-way ANOVA, followed by Sidak’s Multiple Comparison as post-hoc test. Unpaired non-parametric Mann–Whitney test was also performed for comparison between two Gaussian populations, when applicable, as described in figure legends. Significance was defined as *p* < 0.05.

## 3. Results

### 3.1. Synaptic and Extrasynaptic GluN2B-Composed NMDAR Are Altered in Early HD Stages

The two most common non-obligatory NMDAR subunits, GluN2B and GluN2A, predominate in the striatum [19] and have differential roles in synaptic plasticity and NMDARs function in adult cortex, as well as different patterns of expression at PSD [20].

**Figure 1:**
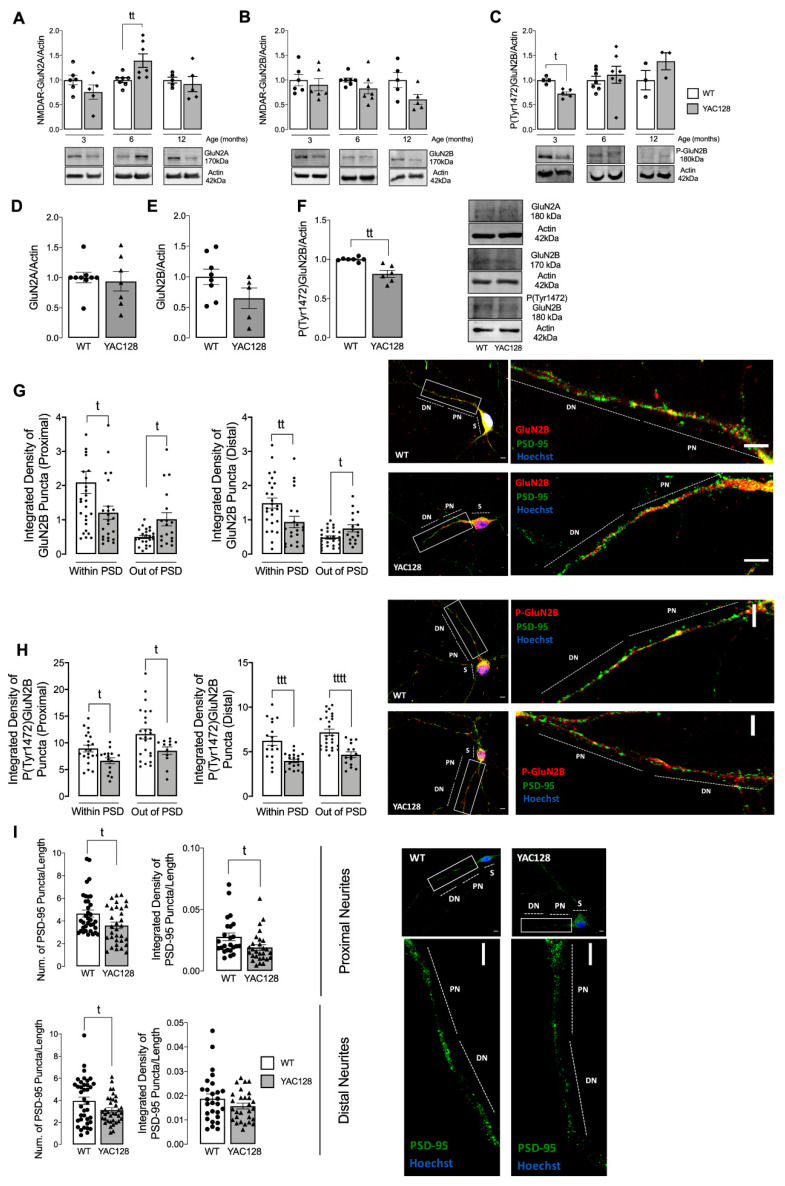
Altered GluN2A- and GluN2B-composed NMDARs levels in HD models. GluN2A/Actin (**A**,**D**)**,** GluN2B/Actin (**B**,**E**) and P(Tyr1472)GluN2B/Actin (**C**,**F**) were analyzed by Western blotting in total extracts obtained from striatal tissue from WT and YAC128 mouse brain (**A**–**C**) and total extracts obtained from primary striatal mouse YAC128 and WT neurons (**D**–**F**)**.** Data are expressed in arbitrary units relative to Actin as the mean ± SEM of 5 to 7 independent experiments. The levels of GluN2B (**G**) and P(Tyr1472)GluN2B (**H**) in co-localization with PSD-95, or out of PSD, as well as, the levels and number of puncta of PSD-95 (**I**), were evaluated by immunocytochemistry, in proximal (from soma to 50 μm) and distal (more than 50 μm) neurites, using confocal microscope and Image J software in YAC128 vs. WT striatal neurons. Confocal images were obtained with a 63× objective in confocal microscope Zeiss LSM 710 (scale bar: 10 μm). Data are presented as the mean ± SEM of 4 independent experiments considering ~5 to 8 cells per condition per culture. Figure legend: S-Soma; PN-Proximal Neurites; DN- Distal Neurites; Statistical analysis: ^t^ *p* < 0.05, ^tt^ *p* < 0.01, ^ttt^ *p* < 0.001, and ^tttt^ *p* < 0.0001 versus control conditions, by nonparametric Mann-Whitney test.

Thus, we initially determined the relative levels of GluN2B and GluN2A NMDARs in striatum from YAC128 mouse model at 3, 6, and 12 months of age (Figure 1A–C). Total GluN2A levels were significantly increased at 6 months of age, but not at 3 or 12 months of age in the striatum of YAC128 mice (Figure 1A), whereas total GluN2B levels were not significantly altered at 3–12 months of age (Figure 1B). Additionally, no significant changes were observed in total levels of GluN2B in soma, proximal or distal neurites from YAC128 striatal neuros, compared with WT neurons (Figure S1).

GluN2B phosphorylation at Tyr1472 by SKF is associated with enrichment of synaptic NMDAR [6]. We observed a significant decrease in Tyr1472 phosphorylation of GluN2B subunit in the striatum of 3-month-old YAC128 mice, a relative presymptomatic stage compared with WT mice (Figure 1C). Similar results were observed in primary striatal neurons from YAC128 mice (Figure 1D–F), which may suggest that in early HD stages NMDARs’ retention at the synapse is altered, potentially contributing for modified synaptic function in HD.

To confirm these results, we analyzed co-localization of total and phosphorylated levels of GluN2B-containing NMDARs with PSD or non-PSD sites. As described in other published data [5], in the present paper we defined PSD or non-PSD sites through the co-localization or not with PSD-95, respectively, in proximal (<50 μm) or distal (>50 μm) neurites in primary striatal neurons, as identified in [15]. In accordance with published data, YAC128 mouse striatal neurons showed reduced GluN2B-NMDARs levels at the synapse and augmented in the non-synaptic sites (Figure 1G), both in proximal and distal neurites. Tyr1472 phosphorylation of GluN2B subunit was reduced both in PSD and non-PSD compartments in HD neurons (Figure 1H), indicating that this NMDARs subunit is less phosphorylated. Additionally, primary striatal neurons showed reduced PSD-95 levels and puncta (Figure 1I), which suggests decreased PSD number and potential synaptic pruning in HD striatal neurons.

### 3.2. Fyn Total and Phosphorylated Levels Are Reduced in PSD of HD Neurons

**Figure 2:**
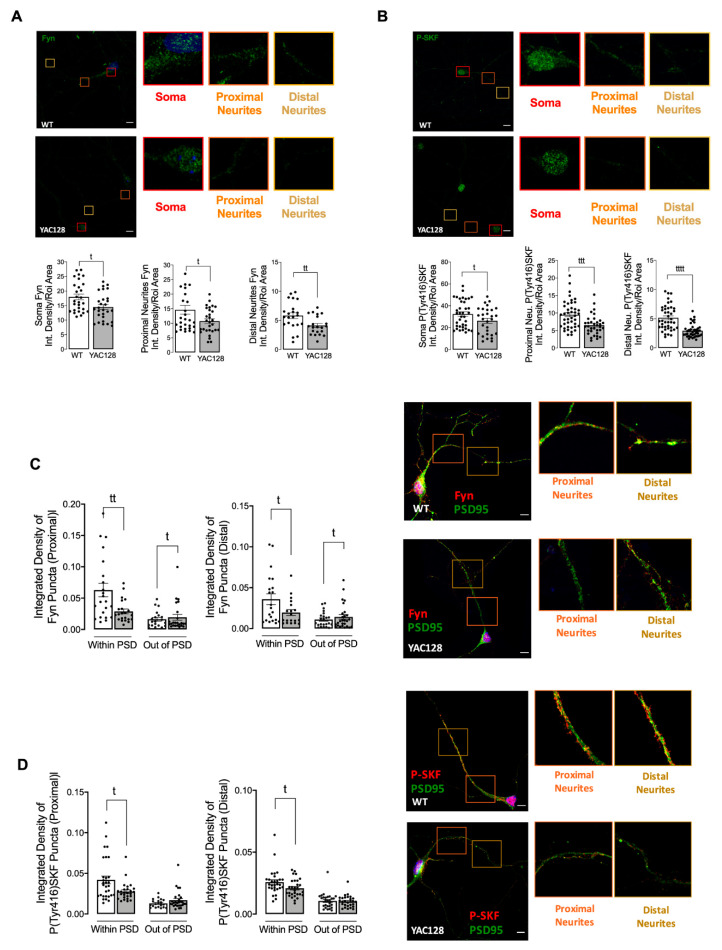
Reduced total and phosphorylated Fyn levels in HD synapses. The total levels of Fyn (**A**) and P(Tyr416)SKF (**B**), as well as, the levels of Fyn (**C**) and P(Tyr416)SKF (**D**) in co-localization with PSD-95, or out of PSD were evaluated by immunocytochemistry in proximal and distal neurites, using confocal microscope and Image J software in YAC128 vs. WT striatal neurons. Confocal images were obtained with a 63× objective in confocal microscope Zeiss LSM 710 (scale bar: 10 μm). Data are presented as the mean ± SEM of 4 independent experiments analyzing 5 to 8 cells per condition per culture. Statistical analysis: ^t^ *p* < 0.05, ^tt^ *p* < 0.01, ^ttt^ *p* < 0.001, and ^tttt^ *p* < 0.0001 versus control conditions (nonparametric Mann-Whitney test).

Fyn play a relevant role in regulating dendritic spine and synapse formation [21,22]. Furthermore, Fyn regulates learning and memory through phosphorylation of NMDAR subunits, playing an important role in synaptic plasticity [23]. Considering that Fyn phosphorylation of GluN2B at Tyr1472 is associated with enrichment of synaptic NMDARs, when compared with extrasynaptic membrane receptors [6], and we recently observed diminished Fyn in several HD models [15], we further analyzed Fyn total and phosphorylated levels at Tyr416, the latter indicating SKF activation since all the family members share the C-terminal. Firstly, we confirmed reduced Fyn total and phosphorylated/active levels in different neuronal sections, from soma to proximal and distal neurites in YAC128 primary striatal neurons (Figure 2A,B). Considering SKF role in NMDARs regulation, we further assessed Fyn total and phosphorylated levels in PSD and non-PSD portions in proximal and distal neurites of primary striatal neurons. As shown in Figure 2C,D, total and phosphorylated Fyn levels are significantly reduced in HD PSD. Taking into account that inhibition of SKF activity with PP2 decreased NMDAR subunits in synaptic and extrasynaptic membranes [6], reduced Fyn levels and activity are apparently related with reduced synaptic GluN2B Tyr1472 phosphorylation (Figure 1H). These data suggest that Fyn reduced levels and activity in PSD may contribute to altered NMDARs phosphorylation and potentially their function.

### 3.3. Constitutive Active SKF Reestablishes GluN2B-Composed NMDAR Levels and Activity in PSD

**Figure 3:**
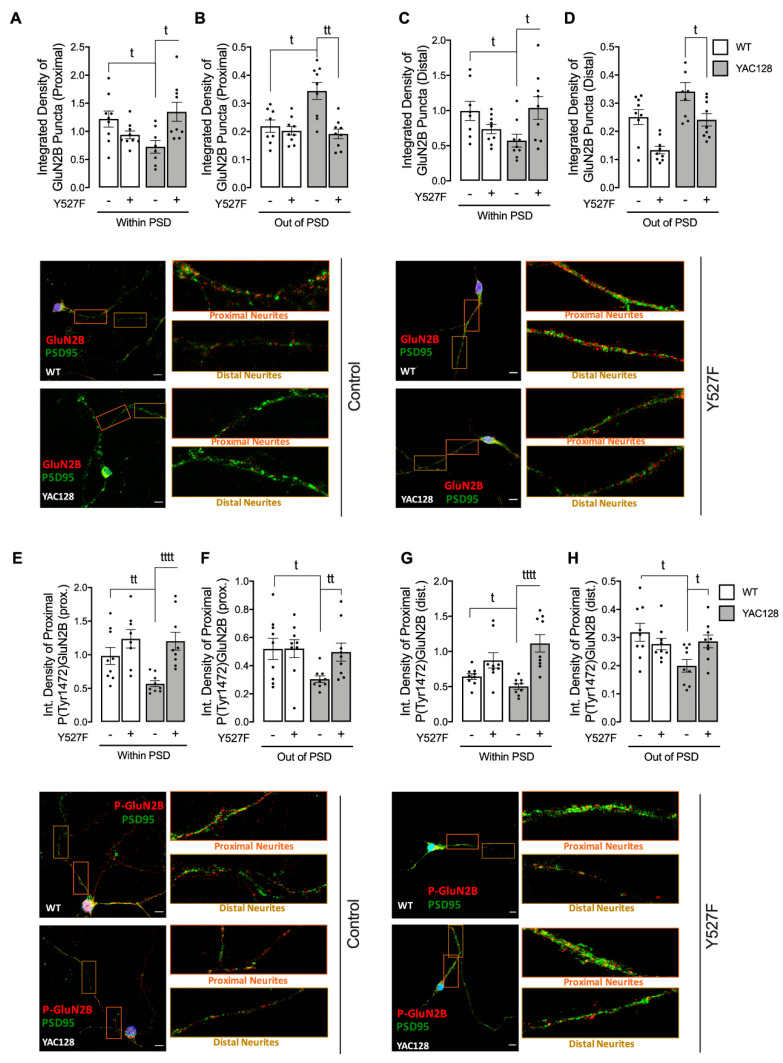
Overexpression of activated SKF restores GluN2B-NMDARs total and phosphorylated levels in PSD sites in HD. The levels of GluN2B (**A**–**D**) and P(Tyr1472)GluN2B (**E**–**H**) in co-localization with PSD-95, or out of PSD, were evaluated by immunocytochemistry, in proximal and distal neurites, using confocal microscope and Image J software in WT vs. YAC128 striatal neurons. Confocal images were obtained with a 63× objective in confocal microscope Zeiss LSM 710 (scale bar: 10 μm). Data are presented as the mean ± SEM of 4 independent experiments considering 5 to 8 cells per condition per condition. Statistical analysis: ^t^ *p <* 0.05, ^tt^ *p* < 0.01, and ^tttt^ *p* < 0.0001 versus control conditions (nonparametric Mann–Whitney test).

Considering reduced Fyn levels and activation within PSD and the observation that Fyn-mediated phosphorylation of GluN2B at Tyr1472 is related with enrichment of synaptic NMDARs [6], we evaluated the influence of SKF proteins on NMDARs localization and function in HD cells. GluN2B-composed NMDARs total and phosphorylated levels were assessed in striatal primary neurons from YAC128 and WT mice, both in PSD and non-PSD sites following transfection with a constitutively active form of the SKF, SKF^Y527F^ (Figure 3). The SKF^Y527F^ mutation enables a mutationally activated form by locking SKF proteins into the open conformation [24]. Importantly, expression of SKF^Y527F^ in YAC128 primary striatal neurons restored GluN2B Tyr1472 phosphorylated levels in both PSD and non-PSD compartments (Figure 3E–H) and GluN2B total levels in PSD, while decreasing GluN2B total levels in a non-PSD compartment (Figure 3A–D) in proximal (Figure 3A,B,E,F) and distal (Figure 3C,D,G,H) neurites. Additionally, no significant differences were observed in total GluN2B levels, in proximal and distal neurites from YAC128 and WT striatal neurons, before and after transfection with SKF^Y527F^ plasmid (Figure S2). These data indicate that augmented SKF activity is important for normal GluN2B phosphorylation and possible synaptic enrichment in HD.

**Figure 4:**
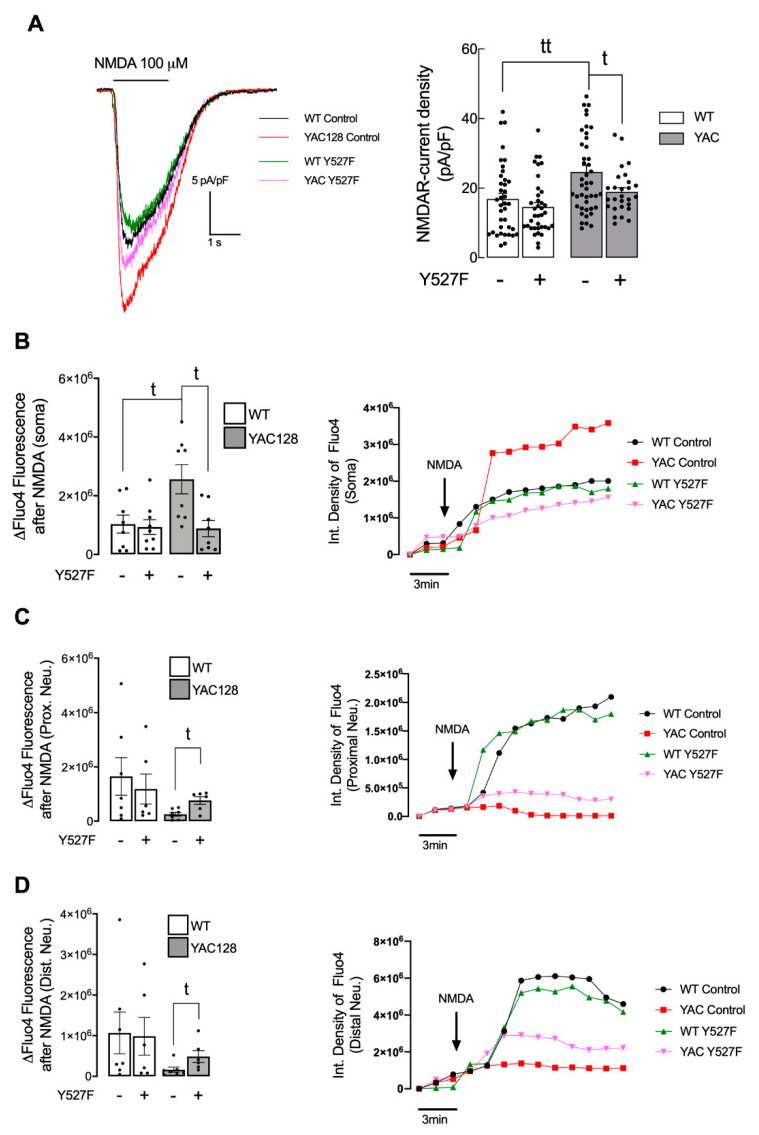
Overexpression of activated SKF partially restores NMDARs currents levels in HD neurons. (**A**) Representative traces of NMDA (100 μM)-induced inward currents (−60 mV holding potential; with 10 μM glycine; without Mg^2+^) in striatal neurons from wild-type (WT) and YAC128 mice showing a higher NMDA-induced current density in YAC128 mice-derived neurons vs. WT-derived neurons, partially restored after expression of SKF^Y527F^, as quantitatively summarized in the histogram. Data are expressed as the mean ± SEM of peak NMDA-induced current density (pA/pF). Statistical analysis: ^t^ *p* < 0.05, ^tt^ *p* < 0.01 by unpaired Student’s *t*-test. (**B**–**D**) Intracellular Ca^2+^ levels were measured using Fluo4 fluorescent dye, in soma (**B**), proximal neurites, (**C**) and distal neurites (**D**) units of fluorescence were monitored before (3 min) and after (15 min) exposure to 100 μM NMDA, in medium without Mg^2+^ and supplemented with glycine (20 μM) and serine (30 μM) in primary striatal neurons from WT and YAC128 mice, using fluorescence microscopy. Data are presented as the mean ± SEM of 4 independent experiments considering ~3 wells/condition. Statistical analysis: ^t^ *p* < 0.05, compared with WT control or YAC128 control (nonparametric Mann-Whitney test).

To support the hypothesis that HD-mediated SKF reduced levels influence the presence and function of NMDARs in the synapse, we evaluated the NMDARs function in soma, proximal, and distal neurites (Figure 4). In accordance with previous published data [5], we observed augmented NMDAR-mediated current density (Figure 4A), and activity, as observed by NMDAR-dependent Ca^2+^ entry (Figure 4B) in the soma of YAC128 striatal neurons. This augmented NMDAR-mediated currents and Ca^2+^-entry were partially reduced after SKF^Y527F^ transfection to levels similar to WT neurons, which suggests that augmented NMDARs occur due to augmented extrasynaptic NMDARs function. On other hand, in proximal (Figure 4C) and distal (Figure 4D) neurites, we observed reduced NMDARs activity in YAC128 striatal neurons, which was reestablished after expression of SKF^Y527F^, augmenting NMDA-induced Ca^2+^-entry. Altogether, these data suggest that altered PSD number and reduced SKF levels and function in HD proximal and distal neurites may result in altered established synapses and reduced NMDARs activity. Of relevance, altered NMDARs activity in proximal and distal neurites can be restored by augmenting active SKF levels. As such, these results suggest that restoration of active SKF reduces extrasynaptic GluN2B-composed NMDARs, augmenting GluN2B-NMDARs at PSD and thus restoring normal NMDAR-dependent Ca^2+^ entry.

### 3.4. Restoration of Active SKF Enhances CREB Activation and Reduces Apoptotic Pathway Activation

**Figure 5:**
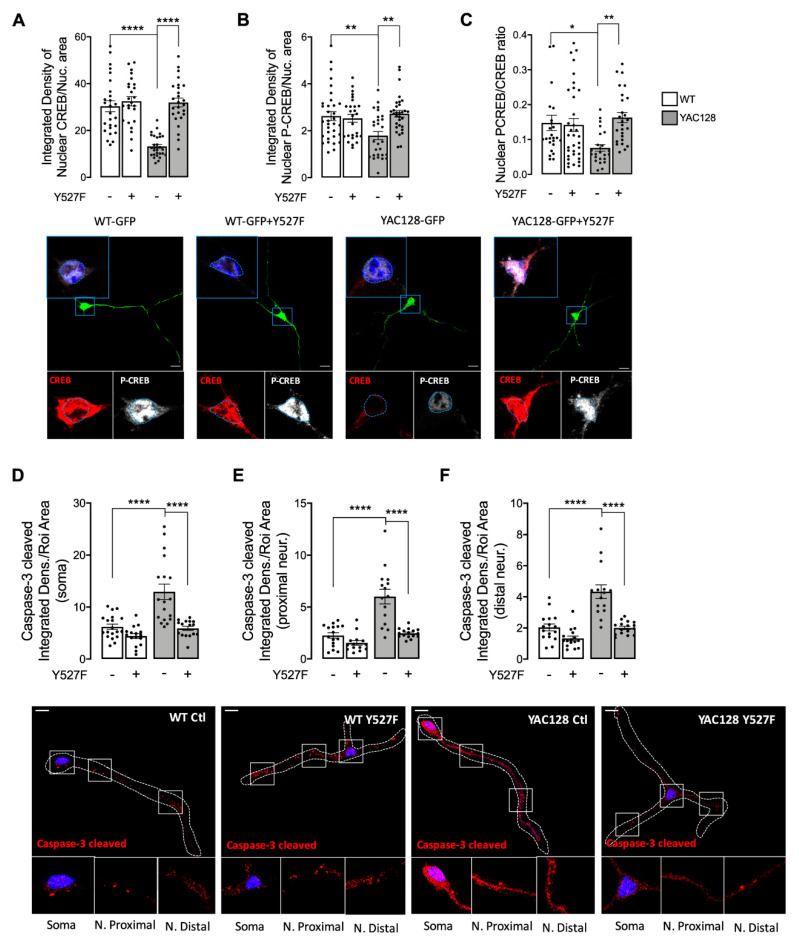
Overexpression of activated SKF restores CREB-activated levels and reduces caspase-3 levels in HD neurons. The nuclear levels of CREB (**A**), P(Ser133)CREB (**B**), and PCREB/CREB (**C**), as well as the levels of cleaved/active caspase-3 (**D**) in soma, proximal, (**E**) and distal (**F**) neurites were evaluated by immunocytochemistry, using confocal microscope and Image J software in WT vs. YAC128 striatal neurons. Confocal images were obtained with a 63× objective in confocal microscope Zeiss LSM 710 (scale bar: 10 μm). Data are presented as the mean ± SEM of 3 to 4 independent experiments considering ~6 wells/condition. Statistical analysis: * *p* < 0.05,** *p* < 0.01, and **** *p* < 0.0001 versus WT or YAC128 (two-way ANOVA, followed by Sidak’s Multiple Comparison as post-hoc test).

Activated CREB promotes the expression of survival-related genes, including BDNF, which has neuroprotective properties and can rescue neurons from NMDAR blockade-induced neuronal death [25]. Additionally, several studies showed that extrasynaptic NMDAR’s activation is associated with cell death pathways, whereas synaptic NMDAR function is associated to augmented CREB phosphorylation and activation, BDNF transcription, and improved antioxidant defenses [26]. Considering restored synaptic NMDAR localization and function after expression of active form of SKF, we next evaluated CREB stimulation and apoptotic pathway activation (Figure 5). As expected, and in accordance with previous published data, we observed reduced CREB protein levels and activation/phosphorylation at Ser133 in YAC128 striatal neuron nuclei (Figure 5A–C). After SKF^Y527F^ expression, CREB protein levels and activation were restored in the nucleus of YAC128 striatal neurons.

Caspase-3 is a cytosolic effector caspase with a central role in apoptosis, specifically being involved in the progression of neurodegenerative disorders [27]. Accordingly, our data showed augmented cleaved/active caspase-3 in YAC128 mouse neurons in soma (Figure 5D), proximal (Figure 5E), and distal (Figure 5F) neurites. Importantly, increased cleaved/active caspase-3 levels were reduced following SKF^Y527F^ plasmid expression, which validates apoptosis modulation (Figure 5D–F).

These results suggest that reestablished SKF levels and activity influence NMDARs presence and function at synaptic membrane, which may activate pro-survival pathways through augmented CREB activation.

## 4. Discussion

NMDARs are important synaptic receptors that contribute to normal synaptic function, cell survival, learning, and memory [28]. Our study shows augmented extrasynaptic GluN2B-composed NMDARs in HD mouse neurons (Figure 1), while expression of a constitutive active form of SKF contributed to reestablish NMDARs localization and activity at PSD (Figure 3). Indeed, reduced Fyn activity in YAC128 PSDs (Figure 2) contributes to reduced GluN2B phosphorylation at Tyr1472, which decreases synaptic NMDAR retention and may facilitate movement to extrasynaptic sites (Figure 4). Apart from restoring NMDARs phosphorylated levels at PSD, enhanced SKF levels and activity promote CREB activation and reduce apoptosis in YAC128 primary striatal neurons (Figure 5).

Previous studies showed that GluN2B-composed NMDARs function and trafficking are altered in HD [5,29,30,31]. Importantly, synaptic or extrasynaptic NMDARs activation are related to survival or apoptosis activation, respectively [32]. Striatal neurons showed faster NMDAR trafficking to the surface membrane induced by mHTT [29], whereas this receptor accumulated at extrasynaptic sites of YAC128 mice at early age [5]. Concordantly, our results evidence a reduction in GluN2B-composed NMDARs levels in PSD, whereas these levels were augmented at extrasynaptic sites is YAC128 mouse striatal neurons. Moreover, in accordance with our data, other studies showed augmented NMDARs currents in HD mouse models [5]. However, this alteration is only measured in HD neuronal soma. In contrast, in proximal and distal neurites, altered PSD number, as well as reduced SKF levels and activity may result in abnormal synaptic function in HD, which may explain the reduction in NMDARs activity. Importantly, altered NMDARs function in proximal and distal neurites can be partially restored by augmenting active SKF levels.

Additionally, GluN2B Tyr1472 phosphorylation levels were decreased both in synaptic and extrasynaptic sites, which is in accordance with previous studies. Gladding and coworkers showed increased synaptic STEP activity in YAC128 striatum, correlated with decreased Tyr1472-GluN2B phosphorylation in YAC128 non-PSD and PSD fractions similar to what we observed in the present study, which facilitates NMDAR movement to extrasynaptic sites, reducing synaptic NMDAR retention [16]. Indeed, GluN2B-composed NMDAR Tyr1472 phosphorylation by SKF is associated with enrichment of synaptic NMDARs [6], indicating that NMDAR lateral diffusion between synaptic and extrasynaptic sites is modulated by Tyr1472 phosphorylation. We previously showed that SKF members, specifically c-Src and Fyn proteins, are reduced in several HD models due to augmented degradation by autophagy [15]. Concordantly with these data, here we show that Fyn total and phosphorylated levels are reduced at both PSD and non-PSD compartments, which may contribute to decrease Tyr1472-GluN2B phosphorylation at PSD and thus reduce synaptic NMDAR retention.

Fyn influence and its fundamental role in PSD has long been recognized. Several studies have previously shown c-Src and Fyn roles in synapse development, plasticity, learning, and memory [22,23]. Takasu and colleagues showed that Ca^2+^ influx mediated by NMDARs activation upon its Tyr1472 phosphorylation by SKF resulted in gene transcription required for the remodeling of synaptic connections in excitatory synapses of primary cortical neurons [33]. In the present study we show, for the first time, that expression of a constitutive active form of SKF reestablishes NMDARs localization and function at PSD in YAC128 mouse striatal neurons, restoring neuronal function.

Decreased synaptic NMDARs and augmented extrasynaptic currents are associated with reduced nuclear CREB activation, reduced neuronal expression of the survival factor BDNF, and caused dysfunctional mitochondria, low energy levels, and cell death in HD mouse striatum, before and after phenotype onset [5]. Extrasynaptic NMDARs activity is linked to cell death signaling cascades due to reduced phosphorylation/activation of CREB [26]. Concordantly with previous studies showing decreased CREB activation in striatum of 1 and 4 months of age YAC128 mice [5], our results show reduced nuclear CREB activation in YAC128 primary striatal neurons (Figure 5A,B). Interestingly, decreased CREB activity and augmented active caspase-3 found in YAC128 striatal neurons were reversed following expression of the constitutive active form of SKF, SKF^Y527F^ (Figure 5), previously shown by our group to be neuroprotective against mHTT-induced mitochondrial dysfunction and enhanced ROS levels [15].

Our study evidence, for the first time, that Fyn protein has an important role in synaptic GluN2B-composed NMDARs phosphorylation and activity, restoring CREB activation and decreasing caspase-3 levels, indicative of a decrease in cell death by apoptosis in HD. Decreased Fyn PSD co-localization correlates with HD-related reduced Tyr1472 GluN2B phosphorylation and augmented extrasynaptic NMDARs currents, as well as decreased CREB activation and cell death. Interestingly, reestablished active SKF levels partially restored NMDARs currents, NMDAR-dependent Ca^2+^ levels, CREB activation, and reduced caspase-3 cleavage. Hence, we describe a potential mechanism involving Fyn restored levels and activity that may constitute a potential HD therapeutic target to promote striatal glutamatergic synaptic function and survival.

## Supplementary Materials

The following supporting information can be downloaded at: https://www.mdpi.com/article/10.3390/cells11193063/s1, Figure S1: Total GluN2B-NMDAR levels in WT and YAC128 striatal neurons. Figure S2: Total GluN2B-NMDAR levels in WT and YAC128 striatal neurons after transfection with Y527F plasmid.

## Abbreviations

5-FDU	5-Fluorodeoxyuridine
BDNF	Brain-derived neurotrophic factor
Ca^2+.^	Calcium ion;
CAG	Cytosine, Adenine, Guanine;
CNS	Central Nervous System;
H_2_O_2._	Hydrogen peroxide;
HD	Huntington’s Disease;
*HTT*	Huntingtin gene;
HTT, Htt	Huntingtin protein (human, mouse);
mHTT, mHtt	Mutant huntingtin protein (human, mouse);
MSNs	Medium spiny neurons;
NMDAR	*N*-methyl-D-aspartate receptor
polyQ	Polyglutamine;
PSD	Post-synaptic density;
ROS	Reactive Oxygen Species;
SKF	Src Kinase Family;
STEP	Striatal-enriched protein tyrosine phosphatase;
YAC	Yeast artificial chromosome
