# Supplementary material for: Restored Fyn Levels in Huntington’s Disease Contributes to Enhanced Synaptic GluN2B-Composed NMDA Receptors and CREB Activity
Source: Cells. 2022 Sep 29;11(19):3063. doi: 10.3390/cells11193063 (PMC9563007; doi:10.3390/cells11193063)
Supplement: Supplementary file 1 [file cells-11-03063-s001.zip › cells-1925584-supplementary.pdf]

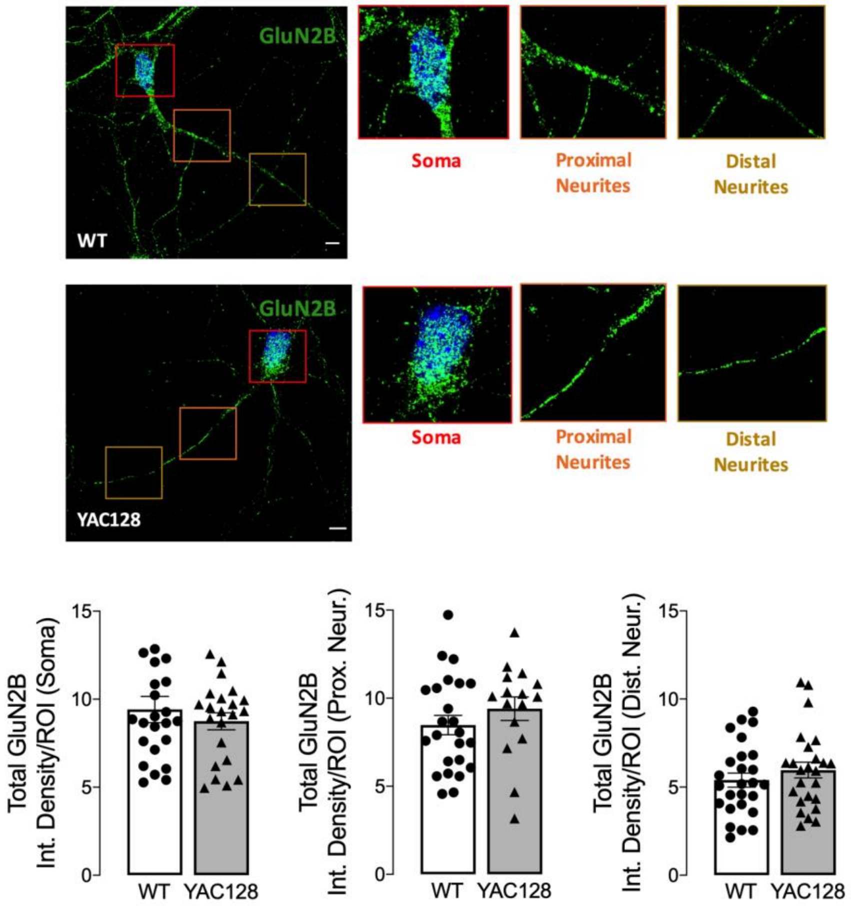

Figure S1: Total GluN2B-NMDAR levels in WT and YAC128 striatal neurons. The total levels of GluN2B were evaluated by immunocytochemistry, in soma, proximal and distal neurites, using confocal microscope and Image J software in YAC128 *vs.* WT striatal neurons. Confocal images were obtained with a 63× objective in confocal microscope Zeiss LSM 710 (scale bar: 10 µm). Data are presented as the mean ± SEM of 4 independent experiments analyzing 5 to 8 cells *per* condition *per* culture.

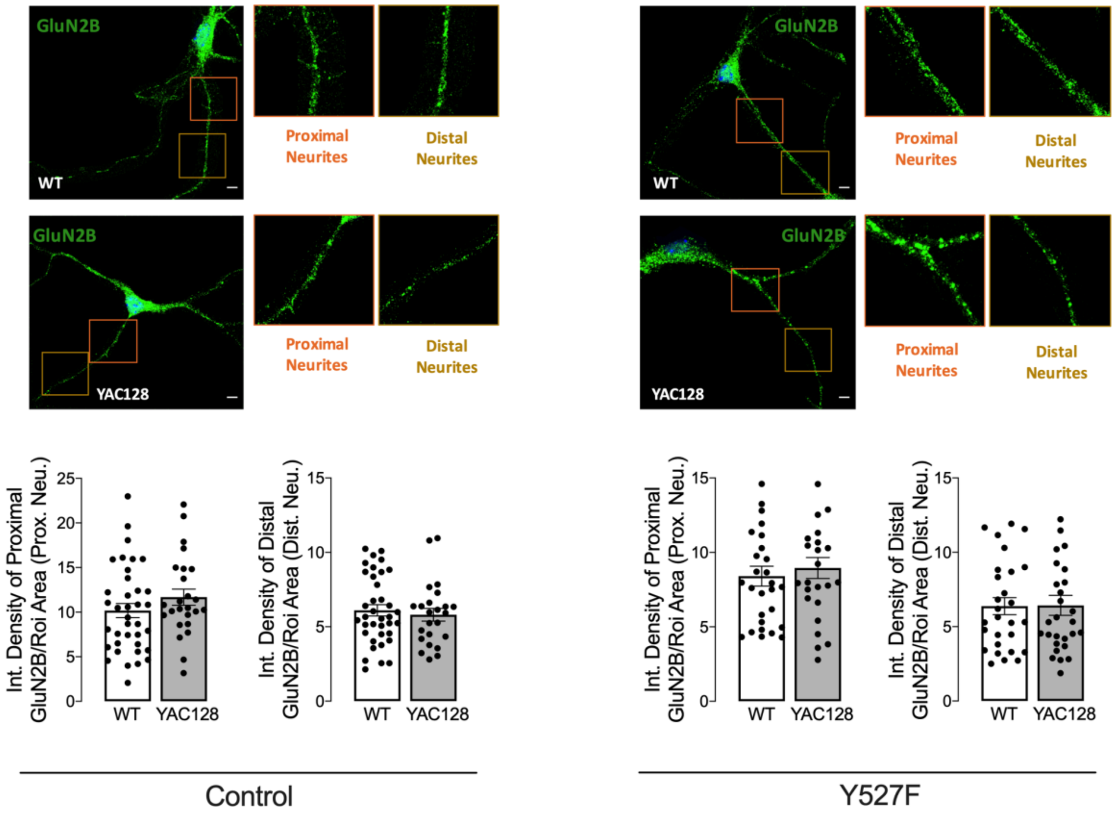

Figure S2: Total GluN2B-NMDAR levels in WT and YAC128 striatal neurons after transfection with Y527F plasmid. The total levels of GluN2B were evaluated by immunocytochemistry, in proximal and distal neurites, using confocal microscope and Image J software in YAC128 *vs.* WT striatal neurons, after transfection with Y527F or control plasmid. Confocal images were obtained with a 63× objective in confocal microscope Zeiss LSM 710 (scale bar: 10 µm). Data are presented as the mean ± SEM of 4 independent experiments analyzing 6 to 8 cells *per* condition *per* culture.
